# Neo-intimal hyperplasia, diabetes and endovascular injury

**DOI:** 10.5830/CVJA-2012-019

**Published:** 2012-10

**Authors:** Deirdré Kruger

**Affiliations:** Department of Surgery, Faculty of Health Sciences, University of the Witwatersrand, Johannesburg, South Africa

**Keywords:** neo-intimal hyperplasia, diabetes, critical limb ischaemia, endovascular stenting, vascular progenitor cells

## Abstract

**Abstract:**

Diabetes is a significant major risk factor for peripheral arterial disease (PAD) and critical limb ischaemia (CLI), the latter which is also the most common cause of amputation in these patients. Revascularisation of the lower extremities of such patients is imperative for limb salvage and has become first-line therapy. However, the incidence of restenosis following endovascular stenting is very high and is largely due to neo-intimal hyperplasia (NIH), the regulation of which is for the greater part not understood.

This article therefore reviews our understanding on the regulation of NIH following stent-induced vascular injury, and highlights the importance of future studies to investigate whether the profile of vascular progenitor cell differentiation, neo-intimal growth factors and lumen diameters predict the severity of post-stent NIH in the peripheral arteries. Results from future studies will (1) better our understanding of the regulation of NIH in general, (2) determine whether combinations of any of the vascular factors discussed are predictive of the extent of NIH postoperatively, and (3) potentially facilitate future therapeutic targets and/or change preventive strategies.

## Abstract

The World Health Organisation projects that diabetes-related mortality will double between 2005 and 2030.^1^ On the other hand, the incidence of peripheral artery disease (PAD), which is usually secondary to atherosclerosis, is expected to rise by 14 to 45% by 2030.^2^ Half of the people with diabetes die of cardiovascular disease, and half of those with diabetes are affected by diabetic nephropathy. This combination of reduced blood flow and neuropathy in the feet of diabetics increases the risk of foot ulcers and eventual limb amputation. Revascularisation in the lower extremities of these patients is imperative for limb salvage, which not only improves the quality of life of these patients but also reduces overall healthcare costs and mortality.

## Diabetes and PAD

From the African Program on Genes in Hypertension (APOGH), the prevalence of diabetes in our local South African setting is reaching 11% (unpublished data; correspondence with APOGH principle investigator, Prof Gavin Norton, 3 May 2011). Diabetes is a significant major risk factor of PAD and critical limb ischemia (CLI), and its incidence and prevalence is on the increase due to the aging of the population.

CLI is defined as the presence of gangrene or non-healing ulceration, rest pain and objective evidence of diffuse pedal ischaemia. The prevalence of diabetes is particularly high in patients with CLI, which is also the most common cause of amputation. Early arterial revascularisation undoubtedly improves the prognosis in these patients.^3^-^7^ CLI progresses to gangrene in 40% of diabetic patients compared with 9% of non-diabetic patients.^8^

A large national vascular registry-based survey from Finland found that diabetes was not an independent risk factor for early postoperative mortality in CLI, as increased morbidity in diabetic patients was associated with old age, male gender, known coronary artery disease, renal insufficiency and, interestingly, urgent surgery.6 Be that as it may, limb salvage rates following superficial femoral artery (SFA) endoluminal interventions are lower for diabetic patients compared to non-diabetics presenting with CLI, despite similar patency and restenosis rates.^9^

## Endovascular interventions of the tibial artery and CLI

Limb salvage not only improves the CLI patient’s quality of life, but also reduces his/her mortality rate and lowers overall healthcare costs.^10^,^11^ As a result, many studies in the last few years have contributed to the change in paradigm of surgical revascularisation for CLI and these endovascular interventions have become first-line therapy for many surgeons at the femoral and popliteal levels.^12^-^14^

Recently, several studies have reported on the efficacy of infra-popliteal interventions for the treatment of CLI.^15^-^17^ However, limited data on the efficacy of tibial artery endovascular intervention (TAEI) in the treatment of CLI, specifically with regard to limb salvage and wound healing, have resulted in recommendations for infra-popliteal disease being rather ambiguous.^18^,^19^ Last year Fernandez *et al*. reported acceptable rates of limb salvage and wound healing following TAEI,^18^ albeit with high rates of re-intervention. Even so, limited data exist and further investigations are required before definitive recommendations for the endovascular treatment of tibial vessel disease can be formulated.

## Neo-intimal hyperplasia and the incidence of restenosis

Within six months of a successful angioplasty, restenosis occurs in up to 55% of patients. Restenosis following angioplasty and stenting is largely due to neo-intimal hyperplasia (NIH) – the thickening of the tunica intima of a blood vessel as a physiological healing response to a reconstructive procedure or endarterectomy. It is currently understood that NIH involves the proliferation and migration of medial smooth muscle cells (SMCs) into a region as it becomes stenotic.^20^

Although therapeutic interventions and continued stent design and coating have been applied, restenosis, even after drug-eluting stent implantation, remains a significant clinical problem.^21^,^22^ Also, therapeutic interventions seem to have preceded the understanding of the biology of the process of restenosis. Moreover, the regulation of NIH is largely not understood and, as a result, advancements in therapeutic interventions are restricted.

The incidence of restenosis a year after coronary angioplasty and bare-metal stenting is approximately 30%,^23^ whereas restenosis a year after carotid artery stenting has been reported as approximately 18%.^24^ A meta-analysis of the incidence of restenosis three years after bare-metal stenting for peripheral artery disease, specifically femoro-popliteal disease, was 61%.^25^ In another study that investigated the efficacy of sub-intimal stent implantation for long, multi-segmental lower limb occlusive lesions, 37% restenosis after approximately one year was reported,^26^ whereas a 17% incidence at two years of re-occlusions and restenosis was reported by a prospective study for sub-intimal angioplasty of the femoro-popliteal or tibial arteries.^27^

While limb salvage, ulcer healing and re-intervention rates are relatively low after below-the-knee endovascular intervention, restenosis rates remain extremely high.^7^ This highlights the importance of understanding the regulation of NIH.

## Therapeutic management of CLI

In CLI patients who are not amenable to surgical intervention for revascularisation, treatment options are very limited. Even though the optimal treatment has yet to be identified, therapeutic angiogenesis is increasingly being used in the non-operative management of CLI. This new strategy includes the administration of growth factors, transcription factors and progenitor cells to induce angiogenesis. Surprisingly, the cytokines used in therapeutic angiogenesis, such as VEGF and FGF, are the also the factors thought to influence NIH following revascularisation.

Similarly, and discussed below, the profile of progenitor cell differentiation in the acute phase post stenting has been suggested as a predictor of restenosis following endovascular intervention. Having said that, clinical trials of cytokine-based therapy in patients not amenable to surgery have produced mixed results, while those using autologous cell transplantation have been much more promising.^28^ Specifically, the safety and efficacy of autologous endothelial progenitor cell therapy has been established from small cohort studies and results from larger trials currently underway will further consolidate this evidence.^29^,^30^

The following sections describe the necessity of investigating the role of cytological and biochemical factors in vessel wall injury and other cardiovascular co-morbidities in an attempt at attenuating NIH following future revascularisation procedures. From this published evidence, it is clear that new strategies in preventing NIH are imperative. Furthermore, a better understanding of the regulation of NIH could facilitate future therapeutic targets.

## Cytological factors and neo-intimal hyperplasia

At the site of stent-induced vascular cell injury, an abundance of cytokines and growth factors are released, and circulating mononuclear cells are mobilised to the site of injury. The local abundance of cytokines and growth factors provides an appropriate environment for cell growth, cell differentiation and cell proliferation.

## Vascular progenitor cells (VPCs)

Circulating mononuclear cells have been implicated in the process of in-stent NIH and VPCs form part of this population of cells.^31^ VPCs differentiate into either endothelial or smooth muscle lineage, depending on the local environment they find themselves in. Only in the last decade have studies suggested the involvement of VPCs in the development of NIH,^32^-^34^ and in 2007, Inoue *et al.* showed a relationship between NIH and the mobilisation of VPCs into the circulation at the acute phase after vascular stent-induced injury.^35^ Moreover, inhibition of NIH by the drug sirolimus is mediated through its potent inhibitory effect on circulating VPCs.^36^

Very recently, Wang *et al.* (2011) showed a strong correlation between the VPC differentiation profile and the severity of post-stent NIH following coronary stenting.^20^ This suggests that VPC differentiation may potentially be a future tool in identifying patients at risk of restenosis after coronary stenting. However, this relationship has not been investigated in any other areas of stent-induced vascular injury. Future studies should investigate the relationship, if any, between the acute-phase VPC differentiation profile and extent of NIH following either femoro-popliteal or tibial artery stenting.

## Biochemical factors and neo-intimal hyperplasia

As mentioned above, an abundance of biochemical factors such as cytokines and growth factors are released at the site of stent-induced vascular cell injury. These factors are important in the regulation of angiogenesis. However, data on their involvement in regulating NIH following lower limb revascularisation are either limited or non-existent.

## Cellular adhesion molecules (CAMs)

CAMs, glycoproteins present on cell surfaces, bind with other cells or the extracellular matrix. The firm adhesion of leukocytes and lymphocytes to endothelial cells are, however, mainly mediated by immunoglobulin CAMs, such as intercellular CAM (ICAM) and vascular CAM (VCAM). Where ICAM binds to integrins present on all leukocytes, VCAM binds to the VLA-4 ligand which is present on lymphocytes, monocytes and eosinophils. Both have been implicated in atherosclerotic lesions.^37^

Activated vascular endothelial cells express elevated levels of CAMs at their surface in the initial phases of atherosclerostic plaque formation. Even though the role of arterial pressure on atherosclerotic plaque development is not fully known, reports have shown high concentrations of circulating ICAM, VCAM, E-selectin and MCP-1 in hypertensive patients.^38^,^39^ Where E- and P-selectin are thought to play a role in atherogenesis, E-selectin participates in the binding of neutrophils and monocytes to endothelial surfaces.

Following vascular injury, CAMs such as ICAM, and E-, L- and P-selectin are responsible for the leukocyte and specifically neurophil accumulation on the injured vessel wall, and various experimental studies have shown their potential role in the initiation and/or development of NIH in both animals and humans.^40^-^42^ Also, Shimazawa *et al*. (2005) showed that the interaction between neutrophil L- and P-selectin with sulfatides, which are ligands for L- and P-selectin, potentially contribute to the development of NIH following vascular injury.^42^

## Nuclear factor kappa B (NF-κB)

NF-κB is a nuclear transcription factor which regulates at least 200 genes involved in cellular proliferation and immune and inflammatory responses. As atherosclerosis is a chronic inflammatory disease, it is not surprising that NF-κB plays an essential role in the development of atherosclerosis. Riou *et al*. (2007) showed a direct link between hypertension and the development of atherosclerosis through the induction of NF-κB.^43^ Rather than affecting SMC proliferation, it has been suggested that NF-κB is involved in apoptotic and inflammatory signalling of vascular SMCs.^44^ Furthermore, NF-κB plays a crucial role in controlling vascular inflammation and NIH.^45^

A recent study by Bu *et al*. (2010) suggested that activation of NF-κB in neo-intimal SMCs following vascular injury induces the expression of a catalytic telomerase reverse transcriptase (TERT). This is one of two core components of the enzyme telomerase, which is responsible for adding DNA sequence repeats to the 3′ ends of DNA strands and is essential to the replicative longevity of vascular cells.^45^

In addition, studies have shown that neutrophil accumulation after vascular endothelial injury contributes not only to the development but also to the initiation of NIH.^46^ Moreover, the pre-operative neutrophil NF-κB status has been suggested as a marker of post-operative organ dysfunction and future studies are warranted to assess the role of NF-κB in neutrophil activation and organ dysfunction following surgery.^47^ The activated neutrophil plays a central role in sepsis (secondary or severe systemic inflammation), which is often present in a patient post vascular intervention. The importance of NF-κB activation in clinical sepsis has not been investigated in patients at risk of lower limb amputations.

## Growth factors

Studies have stressed the role of growth factors on the development of NIH.^48^ Several growth factors stimulate migration and proliferation of medial SMCs into the neo-intima and are released from activated platelets, leukocytes, local endothelial cells and medial SMCs during the cellular proliferation phase of NIH. Furthermore, the release of growth factors by inflammatory cells in the vasculature specifically is of great importance, as atherosclerosis is a chronic inflammatory disease. These growth factors include vascular endothelial growth factor (VEGF), platelet-derived growth factor A and B (PDGF A and B), fibroblast growth factor (FGF) and insulin-like growth factor (IGF). The relationship of the latter factors to NIH is discussed below.

**VEGF:** VEGF is a glycoprotein that stimulates angiogenic and vascular permeability-enhancing activities specific for endothelial cells. The role of the members of the VEGF family in NIH is unknown. Where a number of studies suggest that VEGFs reduce NIH, others propose that they promote restenosis and atherosclerosis. A study in rabbits demonstrated that the strongly angiogenic VEGF-A, VEGF-D and VEGF-D^ΔNΔC^ increased NIH in the carotid artery, which correlated strongly with adventitial angiogenesis.^49^ In fact, several studies have linked adventitial angiogenesis with restenosis.^50^-^52^

VEGF-C-positive macrophages, which were present in the early neo-intima in rats, were later found in the adventitia after its removal, suggesting their involvement in adventitial lymphangiogenesis.^53^ This is of importance as it delivers and activates inflammatory cells that release growth factors which, in turn, promote neo-intimal infiltration and hyperplasia.

Apart from the ischaemic myocardium, the angiogenic effects of VEGF-B are minimal in most organs and many recent studies have described it as a potent neuroprotective factor.^54^,^55^ Surprisingly, it has also been shown to play a role in modulating endothelial fatty acid transportation.^56^

Stefanadis *et al*. (2007) showed a significant reduction in neovessel growth and neo-intimal thickness in New Zealand rats after four weeks of being treated with antibodies specific for VEGF while on a atherogenic diet.^57^ Whether the inhibition of VEGF correlates with a reduced smooth muscle content or has a direct anti-proliferative effect, has yet to be determined. Nevertheless VEGF inhibition showed a possible favourable effect on NIH.

An editorial by Simons (2009) summarised recent evidence suggesting that the role of VEGF in neo-intimal formation occurs in the adventitia.^52^ Specifically, stent-induced damage to the adventitia initiates the local inflammatory response, the production of VEGF, which subsequently induces monocyte chemo-attractant protein-1 (MCP-1) expression in medial SMCs. This results in monocyte accumulation in the adventitia, which also secretes VEGF, to further amplify the cascade that ultimately leads to SMC phenotypic modulation, allowing migration of SMC to the intima. This altered state of SMCs has been established as a critical role in the pathogenesis of NIH.^58^

**PDGF-A, PDGF–B, IGF and FGF:** PDGF, IGF and FGF are important regulators of angiogenesis, and vascular injury increases the availability of many such growth factors. PDGF is a crucial regulator of SMC proliferation and migration. Based on the two polypeptide chains, A and B, different isoforms of PDGF exist. It has been shown that SMCs from injured arteries only secrete PDGF-A. However, such injury exposes these SMCs to platelets within the circulation which not only are a source of PDGF-B, but also, in turn, adhere to the injured artery.

Rat studies have been shown to halt the formation of NIH by specific inhibition of the PDGF receptor, tyrosine kinase.^37^ Similarly, FGF is an important growth factor for SMC, as well as endothelial cell proliferation. Vascular injury releases FGF from the extracellular matrix, after which it is free to bind to its receptors on both SMCs and endothelial cells, promoting cellular proliferation and, as a result, NIH. IGF may play an important role in remodelling the extracellular matrix and, to a lesser extent, increase the ability of PDGF to induce SMC proliferation.

The role of VEGF along with other growth factors (FGF, PDGFs and IGF) involved in stimulating the change required by SMCs to proliferate and migrate to form the neo-intima, as well as its influence on the degree of NIH in the tibial or femoropopliteal artery has not been investigated.

## Conclusion

This research review highlights the need for future studies to investigate whether the profile of VPC differentiation also predicts the severity of post-stent NIH in the femoro-popliteal or tibial artery, as well as the association of neo-intimal growth factors, NF-κB and CAMs with VPC differentiation and formation of the neo-intima. Results from future studies will (1) better our understanding of the regulation on NIH in general, (2) determine whether combinations of any of the vascular factors discussed are predictive of the extent of NIH post operatively, and (3) potentially facilitate future therapeutic targets and/or change preventive strategies.

Specifically, studies from Africa are needed to not only establish and record the incidence of NIH from our local population, as currently, no published data exist, but also to determine which of these factors could predict neo-intimal formation in our population. We also need to determine whether this profiling differs from that of others internationally.
